# Telemedicine in healthcare access during the covid-19 pandemic: a scoping review

**DOI:** 10.11606/s1518-8787.2023057004748

**Published:** 2023-05-11

**Authors:** Mariana Prado Freire, Letícia Gabriela Silva, Ana Ligia Passos Meira, Marilia Cristina Prado Louvison

**Affiliations:** I Universidade de São Paulo Faculdade de Saúde Pública. Departamento de Política, Gestão e Saúde São Paulo SP Brasil Universidade de São Paulo. Faculdade de Saúde Pública. Departamento de Política, Gestão e Saúde. São Paulo, SP, Brasil

**Keywords:** COVID-19, Telemedicine, Telemedicine Emergency Care, Continuity of Patient Care, Health Services Accessibility

## Abstract

**OBJECTIVE:**

Mapping the role of telemedicine in the health access of patients with chronic diseases in continuous care actions (except for covid-19) during the pandemic.

**METHODS:**

This is a scoping review, with an adapted version of the Prisma-Scr methodology and using the Population (patients with chronic diseases), Concept (telemedicine as a health access tool) and Context (covid-19 pandemic) strategy. We searched through the following databases: PubMed, Scopus, Embase, Web of Science, Lilacs and SciELO, resulting in 18 articles at the end of the review. We used the technological, sociocultural and assistance analysis dimensions.

**RESULTS:**

Eighty-eight percent of the analyzed papers posited that telemedicine use to provide care increased during the pandemic. We identified that this use was positively related to the reduction of complications and the absence of physical displacement for care, expanding it to rural areas. Important barriers were presented, most importantly the digital exclusion, language sociocultural barriers, and inaccessibility to technological instruments for disabled people.

**CONCLUSIONS:**

Innovation in care arrangements calls attention to how living labor is important to produce healthcare, using various technologies, and reveals tensions caused by the forces acting on healthcare micro politics. We conclude that, despite important barriers, telemedicine contributed to the care of chronic patients during the covid-19 pandemic.

## INTRODUCTION

The organization and preparation of international health systems changed after the declaration of the Public Health Emergency of International Concern (PHEIC) issued by the World Health Organization (WHO) in January, 2020, due to the global outbreak of coronavirus^1^. Recommendations included supporting fragile health systems, developing immunizers, as well as therapeutic strategies, combating misinformation, strengthening diagnostic mechanisms with an emphasis nor only on isolation, but also transmission prevention, and stimulating the sharing of scientific knowledge and international cooperation.

The first months of viral contamination posed enormous challenges to treating infected patients, overstretching health systems and demanding health services, such as hospitals and outpatient clinics, to change their routine immediately. The concentration of efforts in treating cases of severe acute respiratory syndrome (SARS) caused postponements and cancellation of face-to-face health actions to protect patients from exposure to the virus^2^.

The high case incidence during the pandemic created new strains of SARS-CoV-2, collapsing many health systems, which made it urgent to resume the care of non-covid patients and the chronically ill, offering continuous care^6,7^;. The need to reorganize services, tasks and reinvent ways of doing health was strongly evidenced and considered fundamental^8^. Thus, the technology use gained global prominence in health actions, work and educational activities, as well as to financial and commercial transactions^2^.

The first pandemic confronting reports indicate the monitoring of suspected and confirmed cases by phone or smartphone application, as well as the deployment of telemedicine tools^2^ to guide the general population. These tools served as an initial screening to measure the severity of cases, helping to guide the users’ search for health services, with the objective of prioritizing demand. Countries such as France and the United Kingdom implemented telemedicine actions early, ensuring the compensation of the procedures through the National Health Insurance^9^ and using voice and video resources that also increased the self-care of patients with respiratory diseases^10^.

Even before the emergence of the new coronavirus, several factors contributed to the growth of telemedicine, such as technological advances in communication and information. This a result of the increasing use of high-speed internet and the rise in the number of files in electronic medical records in health services^11^. For Cordioli^12^, telemedicine comprises the service provision related to healthcare in cases where distance is a critical factor, and can be used both for urgent consultations, in the context of covid-19, and routine appointments, given the need of overcoming access barriers, ensuring data protection and providing alternatives to physical examination.

In this context, telemedicine (or telehealth) has different applications, such as teleconsultation, telemonitoring, teleregulation, teleorientation, among others^13^. Currently, the term telemedicine is associated with the terms telehealth and e-Health, with imprecise conceptual distinctions^14^. During the expanded literature search, it was possible to distinguish the use of telemedicine in two large groups: the use of technology as a care arrangement for infected patients and the use of technology as a care arrangement for non-covid patients, a possibility for which this study is interested, regarding access and continuity of care.

This scoping review aims at mapping the contribution of telemedicine to health access of patients with chronic diseases in continuous care actions – non-covid – in the context of the pandemic. The chosen methodology makes it possible to identify the existing literature on the subject, providing elements to analyze the use of telemedicine in the context of covid-19, recognize innovations and new care arrangements, and locate barriers to health access. The analysis of healthcare produced during the pandemic, in the different dimensions, can help us elaborate more balanced public policies and build resilient health systems, with immediate emergency response, and also improve healthcare of chronic patients^15^.

## METHODS

This is a scoping review, which is a literature review modality that has been widely used in mapping the existing literature on a given topic, allowing us to recognize and clarify definitions and conceptual limits^16,17^. In order to do so, we used an adapted version of the Prisma-scr manual^18^, which clusters 22 verification steps concerning the title, abstract, introduction, method and discussion.

We initially used the population, concept and context (PCC) strategy to direct and define the scope of the study, with P (population) = patients with chronic diseases, C (concept) = telemedicine as a tool for access to health and C (context) = covid-19 pandemic. Using the PCC allowed us to formulate our research question “did telemedicine contribute to access to health services for patients with chronic diseases during the covid-19 pandemic?”. This question directed the searches for scientific papers indexed in PubMed, Scopus, Embase, Web of Science, Lilacs and SciELO, selected from the Academic Information Agency of the Universidade de São Paulo with the indexing criterion defined for journals in Public Health^19^. The searches in the databases were carried out between January and March, 2022.

**Figure 1:**
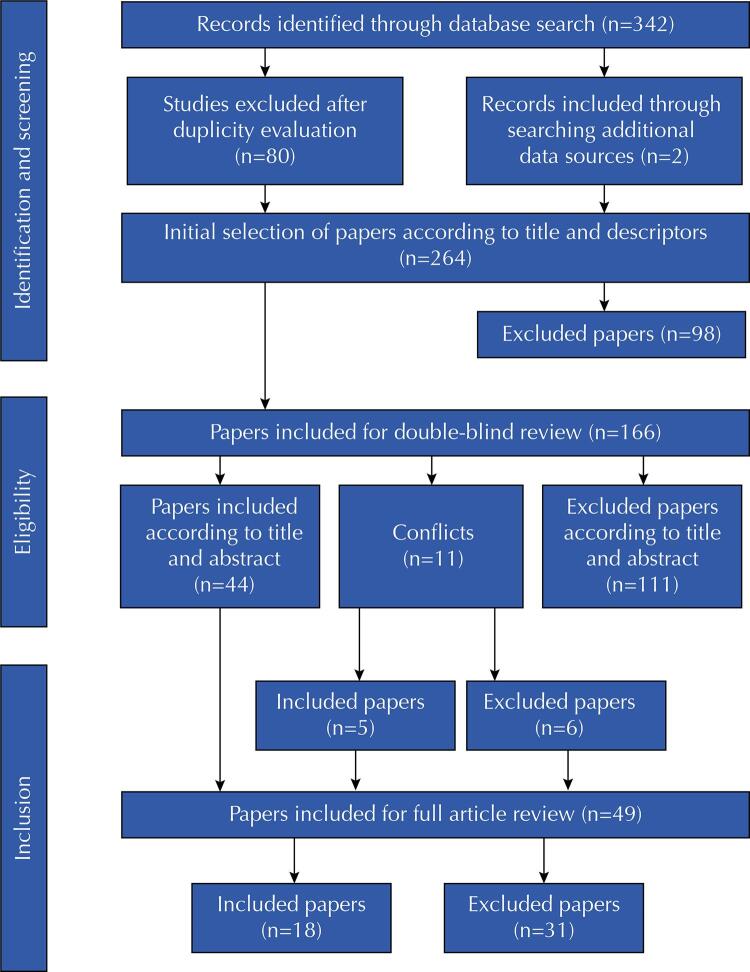
Flowchart of the scoping review inclusion and exclusion process.

The search of scientific papers which integrated the review occurred from the search command-line built with the Health Science Descriptors - DeCs (Telemedicine AND Chronic Diseases AND COVID-19 AND Access to health care), including the period from March, 2020 to March, 2022 for publications in English, followed by double-blind evaluation for the scope assessment stages. All types of scientific papers were included, as well as scientific reviews, without geographical limitation and regardless of publication type. Regarding the results shown in Figure 1, title and keywords were considered in the first analysis and initially included n = 342 papers, 32.16% of which were from PubMed, which retrieved the highest number of results, followed by Web of Science (24.26%) and Scopus (23.39%). The papers were saved in the reference management software Endnote and processed by the platform Rayyan.

In these results, the following exclusion criteria were applied: articles which did not address the use of telemedicine, directed to populations that were not formed by chronic patients, outside the covid-19 pandemic period, discussing specialties that do not fit the chronic criterion and addressing issues related to mental health (n = 98 papers). In the Rayyan platform, duplicates were removed (n = 80 papers) and articles from additional sources were inserted (n = 2 papers), in addition to performing a second double-blind evaluation of the previous results, considering title and abstract (n = 166 papers). At this stage, the inclusion conflicts (n = 11 papers) were sent to new reviewers in double-blind evaluation for final decision, in which n = 5 papers were included.

The analysis included n = 49 papers for full content evaluation; from which, only n = 18 papers were selected for the scope study, because they present elements that can help us answer the research question. We analyzed the following dimensions: technological (type of technology used, resources used, identified innovations), sociocultural (age, income, language) and assistance (type of chronic disease, professionals involved in care, technical and ethical limitations). After extracting the results, they were categorized and discussed by the authors.

## RESULTS

**Figure 2:**
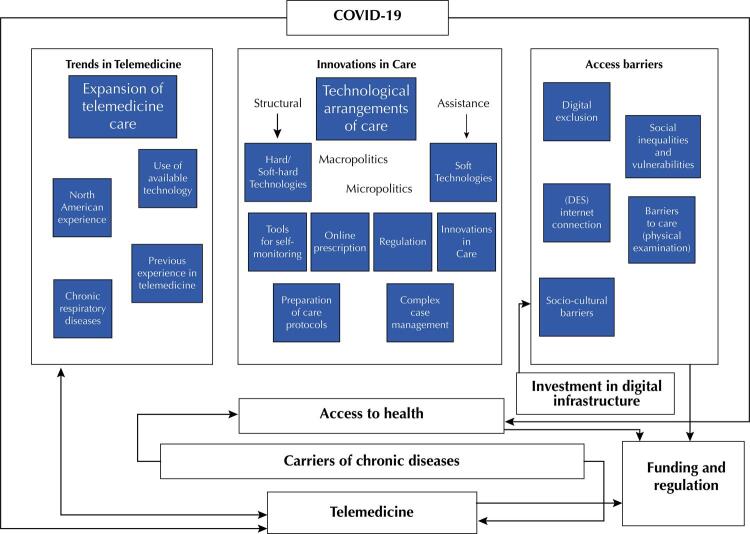
Analytical diagram – telemedicine, chronic diseases and access in the pandemic.

The results identified in the scope of the 18 selected articles are presented in the Chart, containing the characterization of the studies (place of study, publication year, authors, language, description of the study, type of chronic disease studied and type of digital health care technology used). The identified conclusions are presented in Figure 2, an Analytical Diagram including aspects of the analysis carried out by the authors, which will be presented in the discussion.

**Chart:** Articles included in this scoping review.

Title	Publication year	Authors	Place	Object of the study
A qualitative study of high-performing primary care practices during the COVID-19 pandemic^20^	2021	Albert, S. L. and Paul, M. M. and Nguyen, A. M. and Shelley, D. R. and Berry, C. A.	United States	Understand the adaptation of care processes to chronic disease management and preventive care and the future of practices.
Telemedicine in Peru as a Result of the COVID-19 Pandemic: Perspective from a Country with Limited Internet Access^36^	2021	Alvarez-Risco, A. and Del-Aguila-Arcentales, S. and Yanez, J. A.	Peru	It presents the current situation of telemedicine in Peru, showing advances in regulation, cases of successful implementation and current challenges.
New Adopters of Telemedicine During the Coronavirus-19 Pandemic in Respondents to an Online Community Survey: The Case for Access to Remote Management Tools for Individuals with Chronic Obstructive Pulmonary Disease^21^	2021	Boyce, D. M. and Thomashow, B. M. and Sullivan, J. and Tal-Singer, R.	United States	Investigate the adoption of telemedicine, avoidance of the emergency room, and related characteristics of patients with chronic obstructive pulmonary disease (COPD) with and without exacerbations since the beginning of the coronavirus pandemic 2019 (COVID-19).
The COVID-19 pandemic and access to health care in people with chronic kidney disease: A systematic review and meta-analysis^37^	2021	Deng, D. and Liang, A. and Chui, J. N. and Wong, G. and Cooper, T. E.	Revisão sistemática abrangendo estudos de cinco regiões da OMS (49% Região Europeia (EUR), 35% Região da América (AMR), 9% Região do Pacífico Ocidental (WPR), 4% Região do Sudeste Asiático (SEAR) e 4% Região do Mediterrâneo Oriental (EMR)	Assess the effect of the COVID-19 pandemic on access to health care for patients with CRD.
Telemedicine supported strengthening of primary care in WHO South East Asia region: Lessons from the COVID-19 pandemic experiences^31^	2021	Gudi, N. and Konapur, R. and John, O. and Sarbadhikari, S. and Landry, M.	Southeast Asian Countries	Outline the potential role of telehealth in increasing the capacity of Health Systems.
Disparities in Telemedicine Use for Subspecialty Diabetes Care During COVID-19 Shelter-In-Place Orders^22^	2021	Haynes, S. C. and Kompala, T. and Neinstein, A. and Rosenthal, J. and Crossen, S.	United States	Identify patient-level factors associated with the adoption of telemedicine for subspecialty diabetes care during the pandemic.
Perceptions of Telehealth vs In-Person Visits Among Older Adults With Advanced Kidney Disease, Care Partners, and Clinicians^23^	2021	Ladin, K. and Porteny, T. and Perugini, J. M. and Gonzales, K. M. and Aufort, K. E. and Levine, S. K. and Wong, J. B. and Isakova, T. and Rifkin, D. and Gordon, E. J. and Rossi, A. and Koch-Weser, S. and Weiner, D. E.	United States	Identify patient, care partner and nephrologist perceptions of patient centralization, benefits and disadvantages of telehealth compared to face-to-face consultations.
Feasibility of an online platform delivery of pulmonary rehabilitation for individuals with chronic respiratory disease^33^	2021	Lewis, A. and Knight, E. and Bland, M. and Middleton, J. and Mitchell, E. and McCrum, K. and Conway, J. and Bevan-Smith, E.	United Kingdom	Evaluate pulmonary rehabilitation *online* service.
Teleneurology as a Solution for Outpatient Care During the COVID-19 Pandemic^24^	2020	McGinley, M. P. and Ontaneda, D. and Wang, Z. N. and Weber, M. and Shook, S. and Stanton, M. and Bermel, R.	United States	Analysis of *online* consultations in a center specializing in neurology.
Patient and family experience of telehealth care delivery as part of the CF chronic care model early in the COVID-19 pandemic^25^	2021	Solomon, G. M. and Bailey, J. and Lawlor, J. and Scalia, P. and Sawicki, G. S. and Dowd, C. and Sabadosa, K. A. and Van Citters, A.	United States	Determine how people with cystic fibrosis and their families experienced telehealth and assess its quality and acceptability for future care.
Pulmonary Rehabilitation in a Post-COVID-19 World: Telerehabilitation as a New Standard in Patients with COPD^26^	2021	Tsutsui, M. and Gerayeli, F. and Sin, D. D.	United States	Investigate the effects of a supervised telerehabilitation program compared to a conventional supervised pulmonary rehabilitation program.
Where Virtual Care Was Already a Reality: Experiences of a Nationwide Telehealth Service Provider During the COVID-19 Pandemic^27^	2020	Uscher-Pines, L. and Thompson, J. and Taylor, P. and Dean, K. and Yuan, T. and Tong, I. and Mehrotra, A.	United States	Description of the use of telehealth services provided by a well-known company in the United States before and during the covid-19 pandemic. Analysis of the number of virtual visits, their reasons and modifications observed over time.
Narrative Analysis of the Impact of COVID-19 on Patients with Chronic Obstructive Pulmonary Disease, Their Caregivers, and Healthcare Professionals in Italy^32^	2021	Volpato, E. and Centanni, S. and Banfi, P. and D’Antonio, S. and Peterle, E. and Bugliaro, F. and Grattagliano, I. and Piraino, A. and Cavalieri, L. and Pennisi, A. and Danesi, G. and Santoiemma, L. and Marini, M. G.	Itália	Explore how the covid-19 pandemic has impacted quality of care, quality of life, psychological and social factors in people with COPD, their family members and other caregivers and their healthcare professionals and explore how telemedicine in the digital and covid-19 era is described by healthcare professionals and patients and whether it should continue to be included in patient care.
Emerging Alternatives to Conventional Clinic Visits in the Era of COVID-19: Adoption of Telehealth at VCU Adult Cystic Fibrosis Center^28^	2020	Womack, C. and Farsin, R. and Farsad, M. and Chaudary, N.	United States	Present the experience of transition from face-to-face clinical visits to telehealth care in patients with Cystic Fibrosis in response to the covid-19 pandemic. Discussion of the protocol developed and used by the center and the patient’s experience with telehealth in this scenario.
Health Care Providers’ and Professionals’ Experiences With Telehealth Oncology Implementation During the COVID-19 Pandemic: A Qualitative Study^29^	2022	Turner, Kea and Bobonis Babilonia, Margarita and Naso, Cristina and Nguyen, Oliver and Gonzalez, Brian D. and Oswald, Laura B. and Robinson, Edmondo and Elston Lafata, Jennifer and Ferguson, Robert J. and Alishahi Tabriz, Amir and Patel, Krupal B. and Hallanger-Johnson, Julie and Aldawoodi, Nasrin and Hong, Young-Rock and Jim, Heather S. L. and Spiess, Philippe E.	United States	This qualitative study aimed at exploring the experiences of oncology health professionals with the implementation of telehealth during the covid-19 pandemic.
Video Consultation During the COVID-19 Pandemic: A Single Center’s Experience with Lung Transplant Recipients^34^	2021	Kayser, M. Z. and Valtin, C. and Greer, M. and Karow, B. and Fuge, J. and Gottlieb, J.	Germany	Retrospective analysis of video consultations compared with visits *in loco* performed over a 6-week period at a lung transplant center in Germany, using a structured questionnaire and vital signs recording.
Telerehabilitation for Lung Transplant Candidates and Recipients During the COVID-19 Pandemic: Program Evaluation^35^	2021	Wickerson, L. and Helm, D. and Gottesman, C. and Rozenberg, D. and Singer, L. G. and Keshavjee, S. and Sidhu, A.	Canada	Describe the use and satisfaction of providers and lung transplant (LTx) candidates and recipients and functional outcomes after the widespread implementation of telerehabilitation with remote patient monitoring during the first wave of the covid-19 pandemic.
Effect of COVID-19 on Patient Access to HealthServices in Latin America: A Key Informant Survey^30^	2021	Kruse, M. H., Durstine, A. and Evans D.P.	Latin America	Analyze the effects of the pandemic on access to health services for chronic patients through associations that defend patients’ rights.

All papers were published in English, but differed in geographical distribution: n = 10 of the papers were produced in the United States^20^ and n = 1 was a study performed in Latin America^30^. Southeast Asia, on the other hand, was cited in n = 1 paper^31^, and Italy^32^, the United Kingdom^33^, Germany^34^, Canada^35^ and Turkey^36^ also contributed with n = 1 paper each. We identified only one study with a systematic review methodology, which mentioned having considered studies from five regions of the World Health Organization (WHO), with a predominance of articles from Europe^37^.

As for the publication date of the studies, 77% of them were published in 2021, while 16% correspond to the first year of the covid-19 pandemic. In addition, only n = 1 paper was published more recently, in 2022. Regarding the type of study and data source, 44% of the studies are quantitative, with primary data sources and collected through questionnaires applied by telephone or via the internet. Only n = 4 studies used qualitative methods, and n = 2 papers presented narratives as data source; besides, there were systematic reviews, evaluation and mixed methods papers, each of them corresponding to 11% of the total.

Concerning the type of digital health care technology used, 44% of the studies referred to the use of telemedicine^21^. Telehealth was cited in 27% of the analyzed articles ^20,27^, telerehabilitation in 11%^25,32^ and teleneurology^24^, video consultation^34^ and remote monitoring^35^ accounted for 15% of the total. It bears noticing that we mainly considered the technology cited in the study, since some authors used more than one technology.

Chronic respiratory diseases represented 50% of the articles, being the disease group that relied the most on telemedicine, especially chronic obstructive pulmonary disease (COPD) (n = 4 papers) and cystic fibrosis (n = 2 papers). Only 22% of the articles did not delimit the type of chronic disease, characterizing them as chronic diseases in general. We also included studies on diabetes, cancer and chronic neurological diseases, which corresponded to 15% of the total. Half of the identified articles presented the increased use of telemedicine for the care of patients with chronic diseases during the covid-19 pandemic as the main result^20,23^.

The beginning of telemedicine activities was also reported In n = 3 papers^21,26,37^. We identified other results related to the use of telemedicine in the healthcare of chronic patients, such as the improvement of indicators^33^ decrease in complications^36^, increased patient receptivity^29^, video consultations with concrete clinical recommendations / medicine change^34^ and identification of benefits in pre-and postoperative care^35^. On the other hand, there were unfavorable results regarding the use of telemedicine, such as the identification of patients’ inability to use^26^, lower patient engagement^29^, problems with emotional responses and approach to complex issues^29^ and access difficulty, including to electronically prescribed medicines^30^.

The innovations incorporated in the scope of care performed by telemedicine presented structural and assistance characteristics. Among the structural innovations, sending equipment to monitor and measure vital signs (blood pressure measuring device, glucometer, pulse oximeter, home spirometer, among others) to the patients was the most common arrangement, being present in 27% of the studies^20,31,34^.

Blood collection at home^20^, deployment of drive-thru labs^19^ and the delivery of medicine at home^31,36^ were also listed. Other structural arrangements are related to the offered technology itself, such as the availability of e-learning platforms to train patients^33^ and the possibility of using multiple platforms^24^ enabling access for those who have less technological aptitude, in addition to partnerships with University Hospitals and medical schools^31^.

The technological arrangements of care identified as assistance are those invented, adapted or used for the care performed by the health professional or the care chain, through digital technologies. In this sense, it is possible to list prescriptions online^30,31,36^, use telemedicine for comprehensive patient care^28^, including pre-and postoperative care^37^, acting in the regulation and management of complex cases^31^, adoption of detailed pre-consultation protocols^28^ and hybrid care protocols, including face-to-face and online consultations, when necessary^23,26^, and the service performed by multiprofessional team^25^.

Regarding access to health services through telemedicine for patients with chronic diseases during the covid-19 pandemic, 88% of the articles reported access barriers to the use of telemedicine. These were: technological barriers resulting from digital exclusion^20,23,26^, internet access difficulties^23,28,33,35^, connection problems^36^ sociocultural barriers (low purchasing power being the main one)^23,27,36,37^, related to language^23,29^, age^20,34^, disability^29^, the type of health insurance and the telemedicine funding, as well as the assistance access (22%). Among the most important are the limitation in the physical examination of the patient^25,28^, lack of professionals^26^ and aspects regarding specifically the disease or age group, such as hearing problems^23^. All these barriers are mainly related to vulnerable populations, including refugee and immigrant groups^20^.

In addition, aspects that facilitate patients’ access to health care through telemedicine – such as expanding the offer to residents in remote or rural areas (16%)^24,31^, factors related to saving time and resources with commuting^24^ (11%) and the increased involvement of family members and caregivers^20^ (5%) – are pointed out as benefits of the implementation of the remote system. With regard to the future of telemedicine in health systems, the recommendation for the development of guidelines and protocols enabling safe and effective service provision with good digital infrastructure is identified in 83% of studies.

## DISCUSSION

From the mapping and analysis of the data provided by the literature used in this review, we identified the exponential increase in the use of telemedicine and other remote care modalities during the covid-19 pandemic aimed at the care of chronic patients in continuous care. We know that this is an even more comprehensive concept, if we consider here the use of telemedicine forms excluded by the adopted methodological criteria.

After the organization and analysis of the results, we identified three dimensions: trends in telemedicine, innovations in care and access barriers, as shown in Figure 2.

### Trends in Telemedicine

The scope of the selected articles highlights the predominance of studies produced in the United States, especially in scenarios where telemedicine had already been used before the pandemic. The availability of technological structure made it possible to quickly implement^25,32^ these procedures in the USA. In addition, the incorporation of telemedicine in the list of reimbursable procedures by US health plans has served as an incentive since the beginning of the pandemic^20,21^. A study involving a large American telehealth provider also highlighted the increase in demand for care due to chronic diseases and mental health issues, surpassing the search for care motivated by the coronavirus^27^.

On the other hand, in many other locations, such as in China, latent structures gained visibility and could be used in the care of patients because of the health emergency. The authors argue that the structures unveiled in the pandemic should be kept after the mitigation of cases and control of the situation^38^.

We also observed a predominance of papers on the use of telemedicine aimed at the care of patients with chronic respiratory diseases (CRD), a condition that appears among the main causes of morbidity and mortality worldwide. Commonly found, COPD and asthma are among the 20 diseases that disable the most amount of people on a global scale^39^. The use of telemedicine in pulmonology is not recent: Zamith and Gomes^40^ identified studies performed since 1993 containing the association of words “telemedicine” and “lung”. Additionally, the shortage of professionals specialized in pulmonology had already been observed years before the pandemic, and studies that pointed to the use of technological arrangements that could contribute to improving this scenario and guarantee patient access had already been published, such as the described experiences of matrix support and shared care in pulmonology^41,42^.

Besides, the increased demand caused by the pandemic and the potential risk to patients with CRD are also points that contribute to the understanding of the predominance of studies in Pulmonology. Pulmonary telerehabilitation, on the other hand, showed promising results regarding the progression of exercises and improvement of disease indicators^24^, although there are limitations identified in the access of patients^27^.

In Italy, patients with COPD reported receiving twice as many telemedicine visits from pulmonologists as from family doctors^32^. The possibility of providing self-monitoring instruments and the good results the use of equipment at home have demonstrated seem to be factors that give advantages in the monitoring of chronic respiratory diseases, when talking about advances in telemedicine^34^.

### Innovations in Care

The social distancing recommendation adopted by several countries during the pandemic had great adherence among patients with chronic diseases and accentuated difficulties in accessing care, warning about the risk of increased morbidity, disability and avoidable mortality^43^. Brazilian authors emphasize that it is necessary to discuss policies and identify strategies that allow continuity of care, minimizing interruptions and adapting to the new scenario, taking risks of reinforcing or widening inequalities^44,45^.

Based on the conjuncture established by covid-19, the first publications^46,47^ already evidenced the urgency in identifying possibilities for care, encouraging innovations, in an attempt to circumvent the imposed difficulties. Based on the results, telemedicine, generally, presented itself as one of the most important of these innovations, offering powerful mechanisms to act in a scenario of fast-paced contamination^11,21,31,48,49^. Although it was not exactly a new arrangement, telemedicine contributed to the diversification of care, using characteristics such as versatility and broad capacity to reach different populations and health needs. The described innovations demonstrated the importance of offering patient-centered, multilevel, multidisciplinary and continuous care^32^.

The identification of innovative structural arrangements and innovative care arrangements alludes, although in a rudimentary way, to the models of care production and the importance of living labor in the process of care production^50^. Thus, considering that certain arrangements relied more heavily on hegemonic instruments, tools and knowledge – also known as “Hard technologies “ and ”Soft-hard Technologies” – while others were built based on relational aspects, produced in the overlap between health professionals and the patient – known as “Soft technologies”^51^. From this perspective, we identified relevant aspects in each one of them.

The innovative structural arrangements, represented here by sending equipment to the patients’ homes, online prescriptions, drive thru laboratories, among others, raise the issue of telemedicine regulation and funding, inside and outside Brazil. Aspects such as differences in nomenclature and scope, security and protection of patient data, and compensation of services often represent obstacles that must be overcome through defining specific policies and broadly discussing the topic^45,52^.

In Brazil, the regulation in health had been discussed and was moving forward in the Federal Council of Medicine, which published resolution nr. 2.227/2018 at the end of the year, defining important aspects of telemedicine practice. However, institutional disputes motivated its repeal a few days later, conserving the regulatory gap of telemedicine in Brazil^53^. With the pandemic outbreak, the Ministry of Health published the ordinance nr. 467, on March 20, 2020^54^, with temporary provisions for telemedicine actions, supported by Law nr. 13.979, of February 06, 2020^55^, which defined the health emergency in Brazil. The legal device supported the use of telemedicine in Brazil during the emergency, which was declared terminated by the Ministry of Health in the ordinance nr. 913 of April 22, 2022, returning to the Federal Council of Medicine (CFM) the task of regulating telemedicine. More recently, the CFM published resolution nr. 2.314/2022, regulating telemedicine, which still lacks detailed analysis. At the same time, legislative bodies debate a bill on the topic, which expresses the timeliness, urgency and controversy surrounding it.

On the other hand, innovative care arrangements, in turn, portray aspects of the relationships and micro politics of health services^56^. The results found in this review express the tensions experienced in the daily life of services, from bureaucracy to the freedom experienced by health professionals, rooting from unknown situations. The professional performance took place in adverse conditions, outside the comfort zone and with the need to adapt to the unusual scenario. These circumstances made everyday tasks more flexible and enabled the professionals to assume new roles^8^.

In many cases, since there was little regulation and/or a character of exception leveraged by the pandemic, new possibilities of care have emerged, in addition to experiments and incorporation of new protocols^28^. The “temporal window of opportunities”^57^, which opened due to the health crisis, brings complex existential challenges to Public Health in the “post-” pandemic moment. Decentralized caring and integrative practices^42^ are important elements in the analysis of the response to the pandemic.

Finally, the results showed that telemedicine practices have good acceptance rates, both among patients and families and among health professionals and managers^20^. On the other hand, they require in-depth studies regarding cost-effectiveness, quality and user satisfaction; however, the unavailability of data weakens their advancement, regulation, financing and use^58^.

### Access Barriers

The trends and innovations arising from the use of telemedicine for the care of chronically ill patients during the pandemic identified in this review are relevant and offer clues both for policy formulation and for the development of new studies. However, regarding access to health care for the chronically ill using telemedicine, most of the papers included in this review point to numerous barriers^20^. Observing that telemedicine has expanded its borders and is consolidating itself as a care arrangement(s) for chronic diseases^59^ should not be dissociated from the dimension of access and, especially, from the identified barriers. The formulation and implementation of health policies based on technology-mediated care, such as telemedicine and its variations, can both contribute to reducing barriers in health access and highlight inequalities that may compromise the universality of access to health services.

Analyzing the effect that the pandemic has produced on access to health is one of the main current challenges for building resilient health systems. For some authors^60^, the resilience of health systems goes beyond the “fulfillment of the right to health” and encompasses social and economic activities, reproducing the experience of the pandemic. Therefore, early identification of the barriers caused by the use of telemedicine in the health care of chronic patients can prevent the increase of inequities in access to care. In addition, it is essential to include the patient’s dimension, with its diagnostic specificities, in the formulation of policies and protocols^21^.

Although the main challenge regarding access is linked to digital illiteracy (or digital exclusion), the gap evidenced by technology reflects social and health inequalities of the population, whose reduction should guide the construction of quality health systems, with guaranteed access and equity.

However, we also identified factors that can help the patient’s access ^20,24,25,31^, and they can serve as a starting point for policymaking and service implementation. The improvement of the use of digital technologies is central to this discussion and demands political and management efforts in digital infrastructure investment^24,28,31,37^.

We concluded that the increase in telemedicine throughout the covid-19 pandemic presented innovative technological arrangements that, at the same time, collaborated with the expansion of access and with the implementation of this modality of care in the daily life of Health Services. However, issues related to digital exclusion and sociocultural and care conditions were pointed out as access barriers to the use of telemedicine that must be overcome in order to expand, in fact, its use value, caregiver potential and innovation in health systems.

The implementation of specific policies and the elaboration of protocols that guide the work of professionals, particularly for chronic conditions, are important recommendations for incorporating telemedicine as a safe, accessible and care-producing technology for health systems and services around the world.
