# Supplementary material for: Telemedicine in healthcare access during the covid-19 pandemic: a scoping review
Source: Rev Saude Publica. 2023 May 11;57(Suppl 1):4s. doi: 10.11606/s1518-8787.2023057004748 (PMC10185318; doi:10.11606/s1518-8787.2023057004748)
Supplement: Translation

# Translation: Telemedicina no acesso à saúde durante a pandemia de covid-19: uma revisão de escopo

**Authors:** Mariana Prado Freire, Letícia Gabriela Silva, Ana Ligia Passos Meira, Marilia Cristina Prado Louvison

**Affiliations:** I Brasil Universidade de São Paulo. Faculdade de Saúde Pública. Departamento de Política, Gestão e Saúde. São Paulo, SP, Brasil

**Keywords:** COVID-19, Telemedicina, Tele-emergência, Continuidade da Assistência ao Paciente, Acesso aos Serviços de Saúde

## Abstract

**OBJETIVO:**

Mapear a contribuição da telemedicina para o acesso à saúde dos pacientes portadores de doenças crônicas em ações de cuidados continuados (exceto para covid-19) no contexto da pandemia.

**MÉTODOS:**

Trata de revisão de escopo, com adaptação da metodologia Prisma-Scr e utilizou a estratégia População (Pacientes portadores de doenças crônicas), Conceito (Telemedicina como ferramenta de acesso à saúde) e Contexto (Pandemia de covid-19). Foram realizadas buscas nas bases de dados PubMed, Scopus, Embase, Web of Science, Lilacs e SciELO, que resultou em um total de 18 artigos ao final da revisão. As dimensões de análise utilizadas foram tecnológica, sociocultural e assistencial.

**RESULTADOS:**

Entre os artigos analisados, 88% demonstraram o aumento do uso da telemedicina para realização do cuidado durante a pandemia. Identificamos que a utilização da telemedicina esteve relacionada positivamente com a diminuição de complicações e a falta de necessidade de deslocamento físico para o atendimento com ampliação da assistência para áreas rurais. Barreiras importantes foram apresentadas, sendo a exclusão digital e barreiras socioculturais de idioma e falta de acessibilidade dos instrumentos tecnológicos para portadores de deficiência as principais.

**CONCLUSÕES:**

A inovação nos arranjos assistenciais ressalta a importância do trabalho vivo no processo de produção do cuidado, com o emprego de tipos de tecnologias diversos, e expressa tensionamentos em decorrência das forças atuantes na micropolítica do cuidado. Concluímos que, apesar de barreiras importantes, a telemedicina contribuiu para o cuidado dos pacientes crônicos durante a pandemia de covid-19.

## INTRODUÇÃO

A organização e preparação dos sistemas de saúde internacionais ocorreu de maneira diversa após a declaração da Emergência de Saúde Pública de Importância Internacional (ESPII), motivada pelo surto global de coronavírus^1^ e emitida pela Organização Mundial de Saúde (OMS) em janeiro de 2020, em que se recomendava o apoio a sistemas de saúde frágeis e o desenvolvimento de imunizantes e estratégias terapêuticas, combate à desinformação, fortalecimento de mecanismos de diagnóstico com ênfase no isolamento e prevenção da transmissão, estímulo ao compartilhamento de conhecimento científico e à cooperação internacional .

Os primeiros meses de disseminação do vírus imputaram desafios gigantescos em relação ao cuidado dos doentes infectados, exercendo enorme pressão sobre os sistemas de saúde e demandando modificação imediata na rotina dos serviços de saúde como hospitais e ambulatórios. A concentração de esforços no atendimento dos casos de síndrome respiratória aguda grave (SRAG) gerou adiamentos e cancelamento de ações presenciais de saúde, a fim de proteger os pacientes da exposição ao vírus^2^.

A grande incidência de casos durante a pandemia culminou no surgimento de novas cepas do SARS-CoV-2, contribuindo para o colapso de muitos sistemas de saúde, o que tornou urgente retomar o cuidado dos pacientes não-covid e dos doentes crônicos, oferecendo cuidados continuados^6,7^; e a necessidade de reorganizar os serviços, tarefas e reinventar formas de fazer saúde foi fortemente evidenciada e posta como fundamental^8^. Desse modo, a utilização da tecnologia ganhou destaque globalmente nas ações de saúde, atividades laborais e educacionais, além das transações financeiras e comerciais, por exemplo^2^.

Os primeiros relatos de enfrentamento à pandemia indicam a realização de monitoramento dos casos suspeitos e confirmados por telefone ou aplicativo de *smartphone* e a implantação de ferramentas de telessaúde^2^ para a orientação da população em geral, o que serviu como triagem inicial para mensurar a gravidade dos casos, auxiliando na orientação durante a busca dos usuários por serviços de saúde, com o objetivo de priorizar a demanda. Países como França e Reino Unido implantaram ações de telessaúde precocemente, assegurando a remuneração dos procedimentos pelo seguro nacional de saúde^9^ e utilizando recursos de voz e vídeo que também incrementaram o autocuidado de pacientes portadores de doenças respiratórias^10^.

Antes mesmo do surgimento do novo coronavírus, diversos fatores contribuíram para o crescimento da telemedicina, como os avanços tecnológicos das áreas de comunicação e informação, que decorreram do uso crescente da internet de alta velocidade e do aumento do número de registros em prontuários eletrônicos nos serviços de saúde^11^. Para Cordioli^12^, a telemedicina compreende a oferta de serviços ligados aos cuidados com a saúde nos casos em que a distância é um fator crítico, e pode ser utilizada tanto para consultas de urgência, no contexto da covid-19, como para consultas de rotina, diante da necessidade de superar barreiras de acesso, garantir a proteção de dados e fornecer alternativas ao exame físico.

Neste contexto, a telemedicina (ou telessaúde) possui diferentes aplicações, como teleconsulta, telemonitoramento, telerregulação, teleorientação, entre outras^13^. Atualmente, o termo telemedicina é associado aos termos telessaúde e *e-Health*, com distinções conceituais imprecisas^14^. Durante a busca ampliada da literatura foi possível distinguir o uso da telemedicina em dois grandes grupos: o uso da tecnologia como arranjo de cuidado para pacientes infectados e o uso da tecnologia como arranjo de cuidado para pacientes não-covid, possibilidade pela qual este estudo se interessa, no sentido do acesso e continuidade do cuidado.

A presente revisão de escopo tem o objetivo de mapear a contribuição da telemedicina para o acesso à saúde dos pacientes portadores de doenças crônicas em ações de cuidados continuados – não-covid – no contexto da pandemia. A metodologia escolhida possibilita a identificação da literatura existente sobre o tema, fornecendo elementos para analisar o uso da telemedicina no contexto da covid-19, reconhecer inovações e novos arranjos de cuidado e localizar barreiras de acesso à saúde. A análise dos cuidados em saúde produzidos no contexto da pandemia, nas diferentes dimensões, pode colaborar para a elaboração de políticas públicas mais equilibradas e a construção de sistemas de saúde resilientes, com prontidão de resposta a emergências, e ainda no aperfeiçoamento do cuidado de pacientes crônicos^15^.

## MÉTODOS

O estudo trata de uma revisão de escopo (*scoping review*), modalidade de revisão bibliográfica que vem sendo amplamente utilizada no mapeamento da literatura existente sobre determinado tema, colaborando no reconhecimento e esclarecimento de definições e limites conceituais^16,17^. Para o cumprimento das etapas requeridas para a metodologia, utilizou-se de forma adaptada o manual Prisma-scr^18^, que concentra 22 etapas de verificação concernentes ao título, *abstract*, introdução, método e discussão.

Utilizamos, inicialmente, a estratégia denominada de População, Conceito e Contexto (PCC) para direcionar e delimitar o escopo do estudo, sendo P (população) = Pacientes portadores de doenças crônicas, C (Conceito) = Telemedicina como ferramenta de acesso à saúde e C (contexto) = Pandemia de covid-19. O uso do acrônimo contribuiu na formulação da pergunta de pesquisa “A telemedicina contribuiu para o acesso aos serviços de saúde para pacientes portadores de doenças crônicas durante a pandemia de covid-19?”, que direcionou as buscas de artigos indexados nas bases de dados PubMed, Scopus, Embase, Web of Science, LILACS e SciELO, selecionadas a partir da Agência de Informação Acadêmica da Universidade de São Paulo com o critério de indexação definido para periódicos na área de Saúde Pública^19^. As buscas nas bases de dados foram realizadas entre janeiro e março de 2022.

A busca dos artigos que integram a revisão ocorreu a partir da linha de comando de busca construída com os descritores DeCs (*Telemedicine AND Chronic Diseases AND COVID-19 AND Access to health care*), utilizando como critério de inclusão o período de março de 2020 a março de 2022 para publicações em língua inglesa, sucedido de avaliação duplo-cego para as etapas de avaliação de escopo. Foram incluídos todos os tipos de artigos, incluindo revisões, sem limitação geográfica ou tipo de publicação. Em relação aos resultados, demonstrados na Figura 1, na primeira análise foram considerados título e palavras-chave e incluídos, inicialmente, n = 342 artigos, sendo 32,16% da base PubMed, que retornou o maior número de resultados, seguida da Web of Science (24,26%) e Scopus (23,39%). Os artigos foram salvos no software de gerenciamento de referências Endnote e processados pela plataforma Rayyan.

**Figura 1:** Fluxograma do processo de inclusão e exclusão de revisão de escopo.

Nesses resultados, foram aplicados os seguintes critérios de exclusão: artigos que não abordaram o uso da telemedicina, direcionados a populações que não fossem formadas por doentes crônicos, fora do período de pandemia da covid-19, discutindo especialidades que não se enquadram no critério crônico e abordando questões relacionadas à saúde mental (n = 98 artigos). Na plataforma Rayyan, as duplicatas foram removidas (n = 80 artigos) e foram inseridos artigos de fontes adicionais (n = 2 artigos), além de se realizar uma segunda avaliação duplo-cego dos resultados anteriores, considerando título e *abstract* (n = 166 artigos). Nesta etapa, os conflitos de inclusão (n = 11 artigos) foram encaminhados para o parecer de novos revisores em avaliação duplo-cego para decisão final, em que foram incluídos n = 5 artigos.

A análise incluiu n = 49 artigos para avaliação em inteiro teor; desses, apenas n = 18 artigos foram selecionados para o estudo de escopo, em razão de apresentarem elementos que podem auxiliar na resposta à pergunta de pesquisa. Foram analisadas as seguintes dimensões: tecnológica (tipo de tecnologia usada, recursos utilizados, inovações identificadas), sociocultural (idade, renda, idioma) e assistencial (tipo de doença crônica, profissionais envolvidos no cuidado, limitações técnicas e éticas). Após a extração dos resultados, eles foram categorizados e discutidos pelos autores.

## RESULTADOS

Os resultados identificados no escopo dos 18 artigos selecionados estão apresentados no Quadro, contendo a caracterização dos estudos (local da realização do estudo, ano de publicação, autores, idioma, descrição do estudo, tipo de doença crônica estudada e tipo de tecnologia digital do cuidado em saúde empregada). As conclusões identificadas estão apresentados na Figura 2, denominada Diagrama Analítico, que inclui aspectos da análise realizada pelos autores, a ser apresentada na discussão.

**Quadro:** Artigos incluídos nesta revisão de escopo.

Título	Ano da publicação	Autores	Local	Objeto do estudo
A qualitative study of high-performing primary care practices during the COVID-19 pandemic^20^	2021	Albert, S. L. and Paul, M. M. and Nguyen, A. M. and Shelley, D. R. and Berry, C. A.	Estados Unidos	Compreender a adaptação dos processos de cuidado para o gerenciamento de doenças crônicas e cuidados preventivos e o futuro das práticas.
Telemedicine in Peru as a Result of the COVID-19 Pandemic: Perspective from a Country with Limited Internet Access^36^	2021	Alvarez-Risco, A. and Del-Aguila-Arcentales, S. and Yanez, J. A.	Peru	Apresenta a situação atual da telemedicina no Peru, mostrando os avanços na regulação, casos de implementação bem-sucedida e os desafios atuais.
New Adopters of Telemedicine During the Coronavirus-19 Pandemic in Respondents to an Online Community Survey: The Case for Access to Remote Management Tools for Individuals with Chronic Obstructive Pulmonary Disease^21^	2021	Boyce, D. M. and Thomashow, B. M. and Sullivan, J. and Tal-Singer, R.	Estados Unidos	Investigar a adoção da telemedicina, evitar o pronto-socorro e características relacionadas de pacientes com doença pulmonar obstrutiva crônica (DPOC) com e sem exacerbações desde o início da pandemia de coronavírus 2019 (COVID-19).
The COVID-19 pandemic and access to health care in people with chronic kidney disease: A systematic review and meta-analysis^37^	2021	Deng, D. and Liang, A. and Chui, J. N. and Wong, G. and Cooper, T. E.	Revisão sistemática abrangendo estudos de cinco regiões da OMS (49% Região Europeia (EUR), 35% Região da América (AMR), 9% Região do Pacífico Ocidental (WPR), 4% Região do Sudeste Asiático (SEAR) e 4% Região do Mediterrâneo Oriental (EMR)	Avaliar o efeito da pandemia de COVID-19 no acesso aos cuidados de saúde para pacientes com DRC.
Telemedicine supported strengthening of primary care in WHO South East Asia region: Lessons from the COVID-19 pandemic experiences^31^	2021	Gudi, N. and Konapur, R. and John, O. and Sarbadhikari, S. and Landry, M.	Países do Sudeste Asiático	Delinear o papel potencial da telessaúde no aumento da capacidade dos sistemas de saúde.
Disparities in Telemedicine Use for Subspecialty Diabetes Care During COVID-19 Shelter-In-Place Orders^22^	2021	Haynes, S. C. and Kompala, T. and Neinstein, A. and Rosenthal, J. and Crossen, S.	Estados Unidos	Identificar os fatores no nível do paciente associados à adoção da telemedicina para o atendimento da subespecialidade diabetes durante a pandemia.
Perceptions of Telehealth vs In-Person Visits Among Older Adults With Advanced Kidney Disease, Care Partners, and Clinicians^23^	2021	Ladin, K. and Porteny, T. and Perugini, J. M. and Gonzales, K. M. and Aufort, K. E. and Levine, S. K. and Wong, J. B. and Isakova, T. and Rifkin, D. and Gordon, E. J. and Rossi, A. and Koch-Weser, S. and Weiner, D. E.	Estados Unidos	Identificar as percepções do paciente, parceiro de cuidados e nefrologistas sobre a centralização no paciente, benefícios e desvantagens da telessaúde em comparação com as visitas presenciais.
Feasibility of an online platform delivery of pulmonary rehabilitation for individuals with chronic respiratory disease^33^	2021	Lewis, A. and Knight, E. and Bland, M. and Middleton, J. and Mitchell, E. and McCrum, K. and Conway, J. and Bevan-Smith, E.	Reino Unido	Avaliar serviço de reabilitação pulmonar *online*.
Teleneurology as a Solution for Outpatient Care During the COVID-19 Pandemic^24^	2020	McGinley, M. P. and Ontaneda, D. and Wang, Z. N. and Weber, M. and Shook, S. and Stanton, M. and Bermel, R.	Estados Unidos	Análise das consultas *online* em um centro especializado em neurologia.
Patient and family experience of telehealth care delivery as part of the CF chronic care model early in the COVID-19 pandemic^25^	2021	Solomon, G. M. and Bailey, J. and Lawlor, J. and Scalia, P. and Sawicki, G. S. and Dowd, C. and Sabadosa, K. A. and Van Citters, A.	Estados Unidos	Determinar como as pessoas com Fibrose Cistíca e suas famílias vivenciaram a telessaúde e avaliaram sua qualidade e aceitabilidade para futuros cuidados.
Pulmonary Rehabilitation in a Post-COVID-19 World: Telerehabilitation as a New Standard in Patients with COPD^26^	2021	Tsutsui, M. and Gerayeli, F. and Sin, D. D.	Estados Unidos	Investigar os efeitos de um programa de telerreabilitação supervisionado em comparação com um programa de reabilitação pulmonar convencional supervisionado.
Where Virtual Care Was Already a Reality: Experiences of a Nationwide Telehealth Service Provider During the COVID-19 Pandemic^27^	2020	Uscher-Pines, L. and Thompson, J. and Taylor, P. and Dean, K. and Yuan, T. and Tong, I. and Mehrotra, A.	Estados Unidos	Descrição da utilização de serviços de telessaúde fornecidos por empresa conhecida nos Estados Unidos, antes e durante a pandemia de covid-19. Análise do número de visitas virtuais, os motivos das visitas e modificações observadas ao longo do tempo.
Narrative Analysis of the Impact of COVID-19 on Patients with Chronic Obstructive Pulmonary Disease, Their Caregivers, and Healthcare Professionals in Italy^32^	2021	Volpato, E. and Centanni, S. and Banfi, P. and D’Antonio, S. and Peterle, E. and Bugliaro, F. and Grattagliano, I. and Piraino, A. and Cavalieri, L. and Pennisi, A. and Danesi, G. and Santoiemma, L. and Marini, M. G.	Itália	Explorar como a pandemia de covid-19 impactou a qualidade dos cuidados, qualidade de vida, fatores psicológicos e sociais em pessoas com DPOC, seus familiares e outros cuidadores e seus profissionais de saúde e explorar como a telemedicina na era digital e da covid-19 é descrita por profissionais de saúde e pacientes e se deve continuar a ser incluída no atendimento ao paciente.
Emerging Alternatives to Conventional Clinic Visits in the Era of COVID-19: Adoption of Telehealth at VCU Adult Cystic Fibrosis Center^28^	2020	Womack, C. and Farsin, R. and Farsad, M. and Chaudary, N.	Estados Unidos	Apresentação da experiência de transição de visitas clínicas presenciais para cuidados em telessaúde em pacientes com Fibrose Cística em resposta à pandemia de covid-19. Discussão do protocolo desenvolvido e utilizado pelo centro e a experiência do paciente com a telessaúde neste cenário.
Health Care Providers’ and Professionals’ Experiences With Telehealth Oncology Implementation During the COVID-19 Pandemic: A Qualitative Study^29^	2022	Turner, Kea and Bobonis Babilonia, Margarita and Naso, Cristina and Nguyen, Oliver and Gonzalez, Brian D. and Oswald, Laura B. and Robinson, Edmondo and Elston Lafata, Jennifer and Ferguson, Robert J. and Alishahi Tabriz, Amir and Patel, Krupal B. and Hallanger-Johnson, Julie and Aldawoodi, Nasrin and Hong, Young-Rock and Jim, Heather S. L. and Spiess, Philippe E.	Estados Unidos	O objetivo deste estudo qualitativo foi explorar as experiências dos profissionais de saúde de oncologia com a implementação da telessaúde durante a pandemia de covid-19.
Video Consultation During the COVID-19 Pandemic: A Single Center’s Experience with Lung Transplant Recipients^34^	2021	Kayser, M. Z. and Valtin, C. and Greer, M. and Karow, B. and Fuge, J. and Gottlieb, J.	Alemanha	Análise retrospectiva de videoconsultas comparadas com visitas *in loco* realizada durante um período de 6 semanas em um centro de transplante pulmonar na Alemanha, utilizando questionário estruturado e registro de sinais vitais.
Telerehabilitation for Lung Transplant Candidates and Recipients During the COVID-19 Pandemic: Program Evaluation^35^	2021	Wickerson, L. and Helm, D. and Gottesman, C. and Rozenberg, D. and Singer, L. G. and Keshavjee, S. and Sidhu, A.	Canadá	Descrever o uso e a satisfação de provedores e candidatos e receptores de transplante de pulmão (LTx) e os resultados funcionais após a ampla implementação da telerreabilitação com monitoramento remoto de pacientes durante a primeira onda da pandemia de covid-19.
Effect of COVID-19 on Patient Access to HealthServices in Latin America: A Key Informant Survey^30^	2021	Kruse, M. H., Durstine, A. and Evans D.P.	América Latina	Analisar os efeitos da pandemia no acesso aos serviços de saúde para pacientes crônicos através de associações de defesa dos direitos dos pacientes.

**Figura 2:** Diagrama analítico – telemedicina, doenças crônicas e acesso na pandemia.

Todos os artigos foram publicados em inglês, diferindo na distribuição geográfica: n = 10 dos artigos foram produzidos nos Estados Unidos^20^ e n = 1 foi estudo feito na região da América Latina^30^. Já o Sudeste Asiático foi citado em n = 1 estudo^31^, e Itália^32^, Reino Unido^33^, Alemanha^34^, Canadá^35^ e Peru^36^ também contribuíram com n = 1 artigo cada. Identificamos apenas um trabalho com metodologia de revisão sistemática que mencionou ter considerado estudos de cinco regiões da Organização Mundial de Saúde (OMS), com predomínio de artigos da Região Europeia^37^.

Quanto à data de publicação dos estudos, 77% dos estudos foram publicados em 2021, enquanto 16% das publicações correspondem ao primeiro ano da pandemia de covid-19. Além disso, apenas n = 1 artigo foi publicado mais recentemente, em 2022. Em relação ao tipo de estudo e à fonte de dados, 44% dos trabalhos são quantitativos, com fonte de dados primárias e coletados através de questionários aplicados pelo telefone ou via internet. Apenas n = 4 estudos utilizaram métodos qualitativos, sendo que n = 2 artigos apresentaram narrativas como fonte de dados; além disso, houve artigos de revisão sistemática, de avaliação e de métodos mistos, cada um deles correspondendo a 11% do total.

Em relação ao tipo de tecnologia digital de cuidado em saúde utilizada, 44% dos estudos se referiram ao uso da telemedicina^21^. Já a telessaúde foi citada em 27% dos artigos analisados^20,27^, a telerreabilitação em 11%^25,32^ e a teleneurologia^24^, a vídeo consulta^34^ e o monitoramento remoto^35^ representaram 15% do total. Cabe ressaltar que consideramos sobretudo a tecnologia citada no estudo, visto que alguns autores utilizaram mais de uma tecnologia.

As doenças crônicas respiratórias representaram 50% dos artigos, sendo o conjunto de doenças que mais utilizou a telemedicina, com destaque para a doença pulmonar obstrutiva crônica (DPOC) (n = 4 artigos) e a fibrose cística (n = 2 artigos). Apenas 22% dos artigos não delimitaram o tipo de doença crônica, caracterizando-nas como doenças crônicas em geral. Incluímos ainda trabalhos sobre diabetes, câncer e doenças crônicas neurológicas, que corresponderam a 15%. Metade dos artigos identificados apresentaram como principal resultado o aumento do uso da telemedicina para o atendimento de pacientes portadores de doenças crônicas durante a pandemia de covid-19^20,23^.

O início de atividades de telemedicina também foi relatado em n = 3 artigos^21,26,37^. Outros resultados relacionados ao uso da telemedicina no cuidado de doentes crônicos, como a melhora de indicadores^33^, diminuição de complicações^36^, maior receptividade por parte dos pacientes^29^, vídeo consultas com recomendações clínicas concretas/troca de medicamento^34^ e identificação de benefícios no pré e pós-operatório^35^foram identificados. Por outro lado, houve resultados desfavoráveis quanto ao uso da telemedicina, como a identificação de incapacidade de uso por parte dos pacientes^26^, menor engajamento dos pacientes^29^, problemas com respostas emocionais e abordagem de assuntos complexos^29^ e dificuldade de acesso, incluindo aos medicamentos prescritos eletronicamente^30^.

As inovações incorporadas no escopo do atendimento realizado por telemedicina apresentaram características estruturais e assistenciais. Entre as inovações estruturais, o envio de equipamentos de monitorização e medição de sinais vitais (aparelho para medição de pressão, glicosímetro, oxímetro de pulso, espirômetro domiciliar, entre outros) para o domicílio dos pacientes foi o arranjo mais comum, estando presente em 27% dos estudos^20,31,34^.

A coleta de sangue domiciliar^20^, implantação de laboratório funcionando em formato *drive-thru*^19^ e a entrega de medicamento em domicílio^31,36^ também foram listados. Outros arranjos estruturais estão relacionados à tecnologia ofertada em si, como por exemplo a disponibilização de plataformas *e-learning* para o treinamento de pacientes^33^ e a possibilidade do uso de múltiplas plataformas^24^ viabilizando o acesso daqueles que apresentam menor habilidade com o universo virtual, além de parcerias com hospitais universitários e faculdades de medicina^31^.

Os arranjos tecnológicos do cuidado identificados como assistenciais são aqueles inventados, adaptados ou utilizados para o cuidado realizado pelo profissional de saúde ou da cadeia de cuidados, através de tecnologias digitais. Neste sentido é possível listar prescrições *online*^30,31,36^, utilização de telemedicina para cuidado integral do paciente^28^, incluindo cuidados pré e pós-operatórios^37^, atuação na regulação e gestão de casos complexos^31^, adoção de protocolos de pré-consulta detalhados^28^ e protocolos híbridos de atendimento, incluindo consultas presenciais e *online*, quando necessário^23,26^, e o atendimento por equipe multiprofissional^25^.

Em relação ao acesso aos serviços de saúde através da telemedicina para pacientes portadores de doenças crônicas durante a pandemia de covid-19, 88% dos artigos relataram barreiras de acesso ao uso da telemedicina: barreiras tecnológicas decorrentes de exclusão digital^20,23,26^, dificuldades de acesso à internet^23,28,33,35^, problemas de conexão^36^; barreiras socioculturais (sendo o baixo poder aquisitivo a principal delas)^23,27,36,37^, relacionadas ao idioma^23,29^, idade^20,34^, deficiências^29^, relacionadas ao tipo de seguro-saúde e ao financiamento da telemedicina, de acesso assistencial (22%). Entre as mais importantes, estão a limitação no exame físico do paciente^25,28^, a falta de profissionais^26^ e aspectos próprios da doença ou da faixa etária, como, por exemplo, problemas de audição^23^. Todas estas barreiras estão relacionadas principalmente às populações vulnerabilizadas, incluindo grupos de refugiados e imigrantes^20^.

Além disso, os aspectos que facilitam o acesso dos pacientes ao cuidado em saúde através da telemedicina – como a ampliação da oferta para residentes em áreas remotas ou rurais (16%)^24,31^, fatores relacionados à economia de tempo e recursos com o deslocamento^24^ (11%) e o maior envolvimento de familiares e cuidadores^20^ (5%) – são apontados como benefícios da implementação do sistema remoto. No que tange ao futuro da telemedicina nos sistemas de saúde, a recomendação de desenvolvimento de *guidelines* e protocolos que possibilitem a atuação segura e eficaz com boa infraestrutura digital é identificada em 83% dos estudos.

## DISCUSSÃO

A partir do mapeamento e análise dos dados fornecidos pela literatura utilizada na presente revisão, identificamos o aumento exponencial do uso da telemedicina e demais modalidades de cuidado à distância durante a pandemia de covid-19 voltadas para o atendimento de doentes crônicos em cuidados continuados. Sabe-se que o universo é ainda mais abrangente, se consideradas aqui as formas de uso da telemedicina excluídas com base nos critérios metodológicos adotados.

Após a organização e análise dos resultados, três dimensões foram identificadas: tendências em telemedicina, inovações no cuidado e barreiras de acesso, conforme demonstrado na Figura 2.

### Tendências em Telemedicina

O escopo dos artigos selecionados destaca o predomínio de estudos produzidos nos Estados Unidos, especialmente em cenários onde a telemedicina já vinha sendo utilizada anteriormente à pandemia. A disponibilidade de estrutura tecnológica possibilitou rápida implementação^25,32^desses procedimentos no território americano. Somada a isso, a incorporação da telemedicina no rol de procedimentos reembolsáveis por planos de saúde estadunidenses serviu como incentivo desde o início da pandemia^20,21^. Um estudo envolvendo um grande provedor de telessaúde americano destacou ainda o aumento da procura de atendimento para doenças crônicas e questões de saúde mental, superando a busca por atendimento motivada pelo coronavírus^27^.

Por outro lado, em muitas outras localidades, como na própria China, estruturas latentes ganharam visibilidade e puderam ser utilizadas no atendimento dos pacientes em decorrência da emergência sanitária. Os autores defendem que as estruturas desveladas na pandemia não sejam abandonadas após a mitigação dos casos e controle da situação^38^.

A outra tendência observada foi o predomínio de artigos sobre o uso da telemedicina voltada ao cuidado de pacientes com doenças respiratórias crônicas (DRC), condição que aparece entre as principais causas de morbimortalidade em todo o mundo. Comumente encontradas, a DPOC e asma estão entre as 20 doenças que mais causam incapacidade no mundo^39^. O uso de telemedicina em pneumologia não é recente: Zamith e Gomes^40^ identificaram estudos feitos desde 1993 contendo a associação das palavras “*telemedicine*” e “*lung*”. Adicionalmente, a escassez de profissionais especializados em pneumologia já havia sido constatada anos antes, sendo encontrados estudos que apontaram a utilização de arranjos tecnológicos que pudessem contribuir para melhorar esse cenário e garantissem o acesso de pacientes, como as experiências descritas de matriciamento e cuidado compartilhado em pneumologia^41,42^.

Além disso, o aumento da demanda ocasionado pela pandemia e o potencial risco aos pacientes portadores de DRC são também pontos que contribuem para a compreensão da predominância de estudos na área da pneumologia. Já a telerreabilitação pulmonar apresentou resultados promissores em relação à progressão dos exercícios e melhora de indicadores da doença^24^, ainda que haja limitações identificadas no acesso dos pacientes^27^.

Na Itália, pacientes portadores de DPOC relataram que receberam o dobro de atendimentos por telemedicina de pneumologistas que por médicos de família^32^. A possibilidade de disponibilizar instrumentos de automonitoramento e os bons resultados que o uso dos equipamentos em domicílio tem demonstrado parecem ser fatores que conferem vantagens no acompanhamento de doenças respiratórias crônicas, quando fala em avanços em telemedicina^34^.

### Inovações no Cuidado

A recomendação de distanciamento social adotada por diversos países durante a pandemia teve grande adesão entre os portadores de doenças crônicas e acentuou dificuldades de acesso ao cuidado, alertando sobre o risco de aumento da morbidade, incapacidade e mortalidade evitável^43^. Autores brasileiros enfatizam que é necessária a discussão de políticas e a identificação de estratégias que permitam a continuidade do cuidado, minimizando interrupções e adaptação ao novo cenário, sob o risco de reforçar ou ampliar as desigualdades^44,45^.

Com base na conjuntura estabelecida pela covid-19, as primeiras publicações^46,47^ já sinalizavam a urgência na identificação de possibilidades para o atendimento dos usuários, suscitando inovações do cuidado, na tentativa de driblar as dificuldades impostas. Com base nos resultados, a telemedicina, de maneira ampla, se apresentou como uma das mais importantes dessas inovações, oferecendo mecanismos potentes para atuar em um cenário de rápida disseminação^11,21,31,48,49^. Ainda que não fosse um arranjo exatamente novo, a telemedicina contribuiu na diversificação do cuidado, valendo-se de características como versatilidade e ampla capacidade de atuação para atingir diferentes populações e necessidades de saúde. As inovações descritas demonstraram a importância de ofertar cuidados centrados no paciente, multinível, multidisciplinares e continuados^32^.

A identificação de arranjos inovadores estruturais e assistenciais, alude, ainda que de forma rudimentar, aos modelos de produção do cuidado e à importância do trabalho vivo no processo de produção do cuidado^50^. Assim, considerar que determinados arranjos se apoiaram mais fortemente em instrumentos, ferramentas e saberes hegemônicos – também chamados de “tecnologias duras” e “tecnologias leve-duras” – enquanto outros se constituíram levando em conta aspectos relacionais, produzidos no encontro entre o profissional de saúde e o paciente – chamados de “tecnologias leves”^51^. A partir deste olhar, identificamos aspectos relevantes em cada uma delas.

Os arranjos inovadores estruturais, aqui representados pelo envio de equipamentos ao domicílio, prescrições online, laboratórios no formato *drive thru*, entre outros, fazem emergir a problemática da regulamentação e do financiamento da telemedicina, dentro e fora do Brasil. Aspectos como divergências de nomenclatura e escopo, segurança e proteção de dados do paciente e remuneração dos serviços representam, muitas vezes, obstáculos que devem ser superados por intermédio da definição de políticas específicas e ampla discussão da temática^45,52^.

No Brasil, a discussão da regulamentação vinha caminhando a passos largos no Conselho Federal de Medicina, que publicou a Resolução nº2.227/2018 no final do ano, definindo aspectos importantes para a prática da telemedicina. No entanto, disputas institucionais motivaram sua revogação poucos dias depois, conservando o vazio normativo da telemedicina no Brasil^53^. Com a eclosão da pandemia, o Ministério da Saúde publicou a Portaria nº 467, em 20 de março de 2020^54^, com disposições temporárias para ações de telemedicina, amparadas na Lei nº 13.979, de 06 de fevereiro de 2020^55^, que definiu a emergência sanitária no Brasil. O dispositivo legal amparou o uso da telemedicina no território brasileiro durante a vigência da emergência, que foi declarada encerrada pelo Ministério da Saúde na Portaria nº 913 de 22 de abril de 2022, devolvendo ao CFM a tarefa de regulamentar a telemedicina. Mais recentemente, houve a publicação da Resolução CFM nº 2.314/2022, regulamentando a telemedicina, que ainda carece de análise detalhada. Ao mesmo tempo, instâncias legislativas debatem um projeto de lei sobre o tema, o que expressa a atualidade, urgência e controvérsia que o cercam.

Em contrapartida, os arranjos inovadores assistenciais, por sua vez, retratam aspectos das relações e da micropolítica dos serviços de saúde^56^. Os resultados encontrados nesta revisão exprimem as tensões vivenciadas no cotidiano dos serviços, da captura da telemedicina pela burocracia até as experiências de liberdade vivenciadas por profissionais de saúde, forjados inclusive no desconhecido. A atuação profissional se deu em condições adversas, fora da zona de conforto e com a necessidade de adaptação ao cenário inusitado. Estas circunstâncias flexibilizaram as tarefas cotidianas e possibilitaram a assunção de novos papéis^8^.

Em muitos casos, sob a égide da pouca regulamentação e/ou do caráter de exceção alavancados pela pandemia, surgiram novas possibilidades de cuidado, além de experimentos e incorporação de novos protocolos^28^. A “janela temporal de oportunidades”^57^ que se abriu, em decorrência da crise sanitária, traz desafios existenciais complexos no âmbito da saúde pública para o momento “pós” pandemia. As práticas cuidadoras e integrais^42^ descentralizadas são elementos importantes na análise da resposta à pandemia.

Por fim, os resultados demonstraram que as práticas de telemedicina apresentam bons índices de aceitação, tanto entre pacientes e familiares quanto entre profissionais de saúde e gestores^20^. Por outro lado, requerem estudos aprofundados quanto ao custo-efetividade, qualidade e satisfação do usuário; entretanto, a indisponibilidade dos dados fragiliza o seu avanço, regulamentação, financiamento e utilização^58^.

### Barreiras de Acesso

As tendências e inovações advindas do uso da telemedicina para o cuidado de doentes crônicos durante a pandemia identificadas nesta revisão são relevantes e oferecem pistas tanto para a formulação de políticas quanto para a elaboração de novos estudos. No entanto, em resposta à garantia de acesso a cuidados de saúde por doentes crônicos com o uso da telemedicina, a maior parte dos artigos incluídos nesta revisão aponta para inúmeras barreiras^20^. A constatação que a telemedicina expandiu suas fronteiras e vem se consolidando como arranjo(s) do cuidado para doenças crônicas^59^ não deve se dissociar da dimensão do acesso e, principalmente, das barreiras identificadas. A formulação e implementação de políticas de saúde baseadas em cuidados mediados pela tecnologia, como a telemedicina e suas variações, pode tanto contribuir para diminuir as barreiras de acesso à saúde quanto evidenciar desigualdades que possam comprometer a universalidade do acesso aos serviços de saúde.

Analisar o efeito que a pandemia produziu no acesso à saúde é um dos principais desafios atuais para a construção de sistemas de saúde resilientes. Para alguns autores^60^, a resiliência dos sistemas de saúde ultrapassa a “efetivação do direito à saúde” e engloba as atividades sociais e econômicas, reproduzindo a experiência da pandemia. Portanto, identificar precocemente as barreiras causadas em virtude do uso da telemedicina para o cuidado de doentes crônicos pode evitar o aumento de iniquidades no acesso ao cuidado. Além disso, é imprescindível incluir a dimensão do paciente, com suas especificidades de diagnóstico, na formulação das políticas e protocolos^21^.

Ainda que o principal desafio em relação ao acesso esteja vinculado ao analfabetismo digital (ou exclusão digital), o abismo evidenciado pela tecnologia reflete desigualdades sociais e de saúde da população, cuja redução deve guiar a construção de sistemas de saúde de qualidade, com garantia de acesso e equidade.

Todavia, fatores capazes de facilitar o acesso dos pacientes também foram identificados^20,24,25,31^, e podem servir como ponto de partida para elaboração de políticas e implementação de serviços. O aprimoramento do uso de tecnologias digitais é central nesta discussão e demanda esforços políticos e de gestão no investimento em infraestrutura digital^24,28,31,37^.

Conclui-se que o incremento da telemedicina ao longo da pandemia de covid-19 apresentou arranjos tecnológicos inovadores que, ao mesmo tempo, colaboraram com a ampliação do acesso e com a implementação dessa modalidade de atendimento no cotidiano dos serviços de saúde. No entanto, foram apontadas questões relacionadas à exclusão digital e às condições socioculturais e assistenciais como barreiras de acesso para o uso da telemedicina que devem ser superadas para que se ampliem, de fato, seu valor de uso, potencial cuidador e a inovação nos sistemas de saúde.

A implementação de políticas específicas e a elaboração de protocolos que norteiem a atuação dos profissionais, particularmente para as condições crônicas, se apresentam como recomendações importantes no sentido de incorporar a telemedicina como tecnologia segura, acessível e produtora de cuidado para os sistemas e serviços de saúde de todo o mundo.
